# Postoperative dietary management after parotid gland surgery in the absence of high-level evidence: consensus-based guidelines and a stepwise protocol for diet advancement

**DOI:** 10.3389/fsurg.2026.1849468

**Published:** 2026-06-26

**Authors:** Małgorzata Wierzbicka, Cesare Piazza, Dominik Stodulski, Davide Lombardi, Jan Plzak, Jens Peter Klussmann, Miroslav Tedla, Bogusław Mikaszewski, Miquel Quer

**Affiliations:** 1 Department of Otolaryngology, Regional Specialist Hospital Wrocław, Research and Development Centre, Wrocław, Poland; 2 Faculty of Medicine, Wrocław University of Science and Technology, Wrocław, Poland; 3 Department of Procedural Clinical Sciences, Institute of Human Genetics, Polish Academy of Sciences, Poznań, Poland; 4 Unit of Otorhinolaryngology - Head and Neck Surgery, ASST Spedali Civili of Brescia, Brescia, Italy; 5 Department of Surgical and Medical Specialties, Radiological Sciences, and Public Health (DSMC), University of Brescia, School of Medicine, Brescia, Italy; 6 Department of Otolaryngology, Faculty of Medicine, Medical University of Gdańsk, Gdańsk, Poland; 7 Department of Otolaryngology, University Clinical Centre, Gdańsk, Poland; 8 Department of Otorhinolaryngology, Head and Neck Surgery, First Faculty of Medicine, Charles University and University Hospital Motol and Homolka, Prague, Czechia; 9 Department of Otorhinolaryngology, Head and Neck Surgery, Medical Faculty and University Hospital Cologne, University of Cologne, Cologne, Germany; 10 Center for Molecular Medicine Cologne (CMMC), University of Cologne, Cologne, Germany; 11 Department of ENT and HNS, Faculty of Medicine, University Hospital Bratislava, Comenius University, Bratislava, Slovakia; 12 Institute of Cancer and Genomic Sciences, University of Birmingham, Birmingham, United Kingdom; 13 Department of Otolaryngology, Hospital de la Santa Creu i Sant Pau, Barcelona, Spain

**Keywords:** clinical practice guidelines, dietary management, enhanced recovery after surgery, head and neck surgery, parotid surgery, parotidectomy, salivary fistula, sialocele

## Abstract

**Background:**

Despite the ubiquity of dietary advice after parotid surgery, no structured or evidence graded guideline has previously addressed its rationale, timing, or impact on salivary complications. The assumption that “some form of diet is always needed” has persisted largely unchallenged, with heterogeneous habits replacing data driven recommendations.

**Methods:**

An expert panel conducted a structured consensus process informed by systematic reviews of sialocele and salivary fistula prevention, contemporary parotidectomy series, and ERAS based nutritional frameworks for head and neck surgery. Using GRADE methodology and the ESGS/EMSGS anatomical classification, the group developed a surgery stratified, time based dietary advancement protocol.

**Results:**

The proposed algorithm defines decision nodes at postoperative days 0–1, 2–7, week 2, and weeks 3–4, linking dietary texture progression to wound appearance, swelling, pain, and any signs of salivary leakage. The protocol translates subjective, experience based decisions into reproducible criteria adaptable to varied resection extents and patient risk profiles. Recommendations emphasise individualized advancement from low sialagogue, soft regimens toward normal texture as recovery permits.

**Conclusions:**

Standardizing dietary management after parotidectomy reframes nutrition as a modifiable factor in reducing salivary complications while fostering patient understanding and adherence. The consensus highlights the value of early dietitian involvement, clear food lists, and education on mechanical load and salivary stimulation. By codifying a traditionally empirical aspect of care, these guidelines offer clinicians an immediately applicable framework and delineate research priorities for future prospective validation of evidence based dietary care following parotid surgery.

## Introduction

Nutritional management after parotidectomy is a key element of postoperative care, influencing recovery, wound healing, and patient comfort in a substantial global surgical population. International cancer registries estimate 50–55,000 new salivary gland cancers annually, accounting for approximately 0.3% of all malignancies, while overall salivary gland tumour (SGT) incidence, including benign lesions, reaches 0.4–13.5 per 100,000 person-years, with roughly 80% arising in the parotid gland (1). Because surgery remains the standard treatment for most parotid tumours, these figures imply tens of thousands of parotidectomies per year, and many more procedures when benign lesions are included, although precise global operative volumes cannot be calculated due to the lack of systematic registration of benign disease. Existing series from North America and other regions confirm high caseloads but are too heterogeneous to support robust global estimates (2–4).

Despite the SGT burden, postoperative dietary care is guided mainly by local routines and expert opinion (grey literature) rather than high-quality comparative studies (5–9). Most clinicians recommend a soft, bland, nutrient-dense diet to limit mechanical trauma and excessive salivary stimulation, aiming to reduce the risk of salivary fistula/sialocele, wound breakdown and secondary surgical site infection particularly in patients with additional risk factors such as prior irradiation, specific comorbidities or impaired wound healing. However, current dietary protocols are not standardised; they are typically derived from surgeon- or institution-specific materials that emphasise hydration, soft or mechanically altered textures, and avoidance of strong sialagogues, with more restrictive regimens reserved for parotid dissections in which sialocele rates may reach 20%–40% in selected series (8, 10–17). This situation highlights a major evidence gap concerning universal, evidence-based postoperative nutritional guidance and the degree to which diet should be tailored to patient status, tumour characteristics, and resection extent rather than to heterogeneous local practice.

Our central assumption is that structured dietary management is a cornerstone of postoperative care after parotid and other major salivary gland surgery. Although numerous institutional protocols exist, no multicentre expert-panel guideline has yet addressed everyday practice in a systematic, transparent manner. The aim of this study was therefore to bridge the evidence gap by developing consensus-based, structured dietary recommendations and a stepwise protocol for diet advancement after parotid gland surgery, stratified by surgical extent and complication risk, using input from a panel of high-volume parotid surgeons from reference centres.

## Methods

### Study design

This study was conducted as a structured narrative review of the available literature on postoperative dietary management after parotid surgery, complemented by interpretation from a panel of experienced head and neck surgeons. This design was selected because the literature in this field is sparse and heterogeneous, and no prospective or randomized studies were identified that would support the development of recommendations based exclusively on high-level evidence.

### Literature review

A targeted review of the literature was undertaken to identify publications addressing postoperative nutritional management, oral intake, swallowing-related precautions, and diet progression after parotid surgery or comparable salivary gland procedures. Because of the limited number of directly relevant studies, the review also considered adjacent literature and selected non-peer-reviewed sources when these provided practical perioperative information not otherwise available in the peer-reviewed literature; this was considered necessary to map the existing knowledge base in an underexplored area. The scope of the review was intentionally pragmatic rather than systematic. The aim was not to perform a formal evidence synthesis with meta-analytic weighting, but to collect, examine, and organize the limited available information that could inform postoperative dietary recommendations in routine clinical practice.

### Expert panel input

The literature findings were reviewed and discussed by a panel of clinicians with substantial experience in salivary gland surgery. The role of the panel was to interpret the limited published evidence considering real-world surgical practice and to identify areas in which recommendations necessarily relied on clinical expertise because direct supporting evidence was lacking. Based on the literature review and panel discussion, a series of practical statements was formulated regarding postoperative diet advancement. These statements were then organized into a structured step-by-step protocol stratified according to the extent of surgery and the postoperative time course, reflecting the clinical rationale that nutritional tolerance and precautionary needs may differ across these settings.

### Development of recommendations

The recommendations were developed through iterative discussion among the contributing specialists. Rather than using a formalized consensus framework with predefined scoring thresholds or anonymized survey rounds, the final protocol emerged from expert review of the available literature and from collective clinical judgment in areas where the evidence base was insufficient.

To improve interpretability, the revised manuscript distinguishes, where possible, between recommendations supported by published literature and those based primarily on expert opinion. This distinction was considered particularly important given the absence of robust comparative studies in this field.

### Limitations of the methodology

This methodological approach has several limitations. First, as a structured narrative review, it does not provide the procedural rigor of a systematic review and is inherently influenced by the interpretive perspective of the expert panel. Second, the inclusion of selected non-peer-reviewed sources may introduce bias; however, this was deemed justified by the scarcity of peer-reviewed data directly addressing postoperative diet after parotid surgery. Third, because no high-level prospective evidence was available, the proposed protocol should be interpreted as an expert-informed clinical framework rather than a guideline derived from strong evidence-based medicine criteria.

We predefined four domains considered essential for dietary decision-making: (1) patient characteristics (general condition, age, nutritional status, metabolic disorders); (2) tumour and procedure characteristics (tumour size, location, type and extent of surgery); (3) dietary management (diet type, duration, consistency, recommended and contraindicated foods, hydration and supplementation); and (4) perioperative management (compression dressings, chewing-gum use, oral hygiene and dental care, physiotherapy and massage, and pharmacological regimens). The European Salivary Gland Society (ESGS/EMSGS) classification was adopted to standardise anatomical description, dividing the parotid into five levels (I lateral superior, II lateral inferior, III deep inferior, IV deep superior, V accessory) and defining superficial, deep, and total parotidectomy according to the combination of removed levels (18).

A panel of experienced parotid surgeons independently rated the importance of each variable on a 1–5 scale and provided free-text comments, suggestions for additional variables, and priority ratings to guide the final guideline structure. Panel members subsequently scored specific protocol items, including sialagogue timing and diet advancement steps, on 1–9 Likert scales over three iterations, aiming for at least 70% agreement for each level. Where available, statements were cross-checked against observed sialocele and fistula rates stratified by EMSGS extent to ensure biological and clinical plausibility.

## Results

### Burden and preventive context

Recent systematic reviews indicate a pooled incidence of approximately 3% for clinically evident salivary fistula and 4%–5% for sialocele after parotid surgery, with wide inter-study variation and reported ranges up to 20% and over 40%, respectively, in selected cohorts (5, 15, 19, 20). Contemporary single- and multicentre series of benign parotidectomy report combined fistula and sialocele rates of roughly 2%–10%, with higher risks after partial superficial resections, middle or anterior tumour locations, and in patients with unfavourable systemic or local wound-healing profiles (11, 16, 17, 20–24). These data confirm that salivary leakage is one of the most frequent early wound complications after parotidectomy and must be addressed explicitly in perioperative counselling and care pathways.

Current preventive strategies in parotid surgery span three domains: preoperative risk optimisation, meticulous surgical technique, and structured postoperative management. Optimisation of nutritional status, correction of anaemia and endocrine imbalances, and cessation of smoking and alcohol use are associated with lower wound-related morbidity and reduced salivary fistula risk, echoing ERAS-based protocols in major head and neck surgery (5, 19, 20, 25–27). Intraoperatively, gentle handling of the parenchyma and ducts, preservation of vascularity, minimisation of dead space, and watertight, tension-free multilayer closure are consistently linked to lower rates of sialocele and salivary leakage, whereas suction drains should be used selectively and for limited durations rather than routinely (16, 17, 20–23, 27). Postoperatively, early detection of abnormal salivary accumulation, prompt application of compression, and careful control of wound pressure dynamics, supported by close clinical surveillance in the first days after surgery, can prevent fistula maturation and improve functional and aesthetic outcomes (4, 20, 24, 28).

Against this background, we focused on formalising the dietary component of postoperative management, which remains poorly defined in the scientific literature and is largely driven by institutional habits.

### Synthesis of existing dietary recommendations

The targeted review confirmed that English-language literature dedicated specifically to diet after parotidectomy is sparse and that no formal evidence-graded dietary guideline currently exists (Table 1). Most recommendations originate from postoperative information leaflets, institutional protocols, and expert reviews, rather than from prospective comparative studies. Typical advice from high-volume centres involves initial clear fluids followed by transition to a soft or bland diet within 24–48 h, primarily to mitigate early mastication-related discomfort and mechanical irritation of the wound, while emphasising adequate caloric intake and hydration. Patients are commonly advised to avoid hard, sharp, or scratchy foods, very hot beverages, and, for a limited period, strongly acidic, sour, or spicy items and carbonated drinks because of their sialagogue effect.

**Table 1:** Recommendations derived from the formal literature, supplemented by curated internet-based resources (* grey literature).

No	Type	Source
1	*Web guideline	Larian B, Azizzadeh B. Parotidectomy recovery guidelines. Center for Advanced Parotid & Facial Nerve Surgery, Beverly Hills, CA. Available at: https://www.parotidsurgerymd.com/micro-parotidectomy/parotidectomy-recovery-guidelines/. Accessed December 5, 2025.
2	*Patient leaflet	Community ENT. Parotidectomy post operative instructions. Louisville, KY: Community ENT; 2020. Available at: https://communityent.com/wp-content/uploads/2022/06/Copy-of-Parotidectomy-Post-Op.pdf. Accessed December 5, 2025.
3	*Patient leaflet	Otolaryngology Specialists of North Texas. Post-operative instructions for parotidectomy. Plano/Dallas, TX: Otolaryngology Specialists of North Texas; 2019. Available at: https://entkidsadults.com/wp-content/uploads/2019/08/Post-operative-Instructions-for-Parotidectomy.pdf. Accessed December 5, 2025.
4	*Patient leaflet	ENT Center of Utah. Parotidectomy. Post-operative instructions. Murray, UT: ENT Center of Utah; 2021. Available at: https://entcenterutah.com/wp-content/uploads/2021/03/PAROTIDECTOMY.pdf. Accessed December 5, 2025.
5	*Web guideline	Facial Paralysis Institute. Parotidectomy. Beverly Hills, CA: Facial Paralysis Institute. Available at: https://facialparalysisinstitute.com/treatments/parotidectomy/. Accessed December 5, 2025.
6	*Web guideline	MyHealth Alberta. Parotidectomy: what to expect at home. Alberta Health Services; 2024. Available at: https://myhealth.alberta.ca/Health/aftercareinformation/pages/conditions.aspx?hwid=zc2534. Accessed December 5, 2025.
7	*Patient leaflet	Arizona Ear, Nose & Throat Physicians. Parotidectomy post-operative instructions. Available at: https://www.azentsurgery.com/patient-area/parotidectomy-post-op.php. Accessed December 5, 2025.
8	*Web guideline	OncoLink. Surgical procedures: parotidectomy. Abramson Cancer Center of the University of Pennsylvania; 2024. Available at: https://www.oncolink.org/cancers/head-and-neck/treatments/surgical-treatments/parotidectomy. Accessed December 5, 2025.
9	*Patient leaflet	Pristyn Care. Parotidectomy: indications, procedure and recovery. Available at: https://www.pristyncare.com/dehu/surgery/parotidectomy/. Accessed December 5, 2025.
10	*Web guideline	Tibrewal S. Diet plan: foods to eat and avoid after salivary gland surgery. Available at: https://www.drsuniltibrewal.com/diet-plan-foods-to-eat-avoid-after-salivary-gland-surgery/. Accessed December 5, 2025.
11	*Patient leaflet	Mount Sinai Head & Neck Institute. Postoperative instructions: parathyroid surgery. New York, NY: Mount Sinai Health System. Available at: https://www.mountsinai.org/locations/head-neck-institute/postoperative/parathyroid. Accessed December 5, 2025.
12	*Patient leaflet	Cambridge University Hospitals NHS Foundation Trust. Diet and fluid advice for a high-volume stoma or fistula. Cambridge, UK: CUH; 2023. Available at: https://www.cuh.nhs.uk/patient-information/diet-and-fluid-advice-for-a-high-volume-stoma-or-fistula/. Accessed December 5, 2025.
13	*Patient leaflet	Leeds Teaching Hospitals NHS Trust. Dietary advice for patients with a high output stoma or enterocutaneous fistula. Leeds, UK: LTHT; year not stated. Available at: https://flipbooks.leedsth.nhs.uk/LN004214.pdf. Accessed December 5, 2025.
14	*Patient leaflet	Sydney Centre ENT. Post-operative instructions: sialendoscopy. Sydney, Australia: Sydney Centre ENT; 2022. Available at: https://sydneycentreent.com.au/wp-content/uploads/2022/10/Post-Operative-Instructions-Sialendoscopy.pdf. Accessed December 5, 2025.
15	*Web guideline	Larian B, Azizzadeh B. Parotid surgery guidelines. Center for Advanced Parotid & Facial Nerve Surgery; 2024. Available at: https://www.parotidsurgerymd.com/wp-content/uploads/2024/11/parotid-surgery-guidelines.pdf. Accessed December 5, 2025.
16	*Web guideline	Mount Sinai Head & Neck Institute. Postoperative instructions: parotid surgery. New York, NY: Mount Sinai Health System. Available at: https://www.mountsinai.org/locations/head-neck-institute/postoperative/parotid. Accessed December 5, 2025.
17	*Web guideline	ENT of Georgia South. Post-operative instructions for parotid surgery. Available at: https://entgasouth.com/services/neck-throat/post-operative-instructions-for-parotid-surgery. Accessed December 5, 2025.
18	*Web guideline	Lhotský R. Diet after reduction of the saliva gland. 2024. Available from: https://www.radeklhotsky.cz/en/diet-after-reduction-of-the-submandibular-gland/. Accessed December 5, 2025.
19	*Web guideline	Parotid Surgery MD. Parotid surgery guidelines. Beverly Hills: Parotid Surgery MD; 2024. Available from: https://www.parotidsurgerymd.com/wp-content/uploads/2024/11/parotid-surgery-guidelines.pdf. Accessed December 5, 2025.
20	*Web guideline	Parotid Surgery MD. Parotidectomy recovery guidelines. Beverly Hills: Parotid Surgery MD; 2025. Available from: https://www.parotidsurgerymd.com/micro-parotidectomy/parotidectomy-recovery-guidelines/. Accessed December 5, 2025.
21	*Web guideline	Parotid Patient Project. Practical recovery tips for parotid surgery. 2022. Available from: https://parotidpatientproject.org/parotid-education/recovery/practical-recovery-tips.html. Accessed December 5, 2025.
22	*Patient leaflet	Blue Ridge ENT. Post operative care: parotidectomy. 2022. Available from: https://blueridge-ent.com/wp-content/uploads/2022/07/parotidectomy-post-op-care.pdf. Accessed December 5, 2025.
23	*Patient leaflet	Rutgers Cancer Institute of New Jersey. Soft diet 2021. 2021. Available from: https://cinj.org/sites/cinj/files/documents/Soft-Diet-2021.pdf. Accessed December 5, 2025.
24	*Patient leaflet	Royal United Hospitals Bath. Soft diet. 2020. Available from: https://www.ruh.nhs.uk/patients/services/clinical_depts/dietetics/documents/Soft_Diet.pdf. Accessed December 5, 2025.
25	*Patient leaflet	Healthpoint. Examples of soft diet. 2019. Available from: https://www.healthpoint.co.nz/download,007d6e53-3cac-498c-ac36-7e2c9c915a05.do. Accessed December 5, 2025.
26	*Patient leaflet	Memorial Sloan Kettering Cancer Center. Eating guide for puréed and mechanical soft diets. 2015. Available from: https://www.mskcc.org/sites/default/files/node/20288/document/b-137_mech_diet_2015-3.pdf. Accessed December 5, 2025.
27	*Patient leaflet	University Hospitals Birmingham NHS Foundation Trust. Maxillofacial diet advice following surgery for head and neck cancer. 2021. Available from: https://www.uhb.nhs.uk/media/uuup2lbw/pi-maxillofacial-diet-advice-following-surgery-for-head-and-neck-cancer.pdf. Accessed December 5, 2025.
28	*Patient leaflet	Queen Victoria Hospital NHS Foundation Trust. Eating and drinking after head & neck cancer surgery. 2020. Available from: https://www.qvh.nhs.uk/download/patient-information-leaflets/eating-and-drinking-after-head-neck-cancer-surgery/. Accessed December 5, 2025.
29	Original article	Singh A, et al. Comparing the efficacy of 3% NaCl with pressure dressing and oral glycopyrrolate with pressure dressing in the treatment of parotid sialocele in postoperative oral squamous cell carcinoma patients: study protocol of a randomized controlled trial. Cureus. 2024. Available at: https://www.cureus.com/articles/273372-comparing-the-efficacy-of-3-nacl-with-pressure-dressing-and-oral-glycopyrrolate-with-pressure-dressing-in-the-treatment-of-parotid-sialocele-in-postoperative-oral-squamous-cell-carcinoma-patients-study-protocol-of-a-randomized-controlled-trial. Accessed December 5, 2025.
30	Original article	Skorek A, et al. Revision parotidectomy—analysis of indications for the procedure and long-term outcomes. Pol Przegl Chir. 2023;95 (3):1–9. Available at: https://ppch.pl/article/150499/en. Accessed December 5, 2025.
31	Original article	Cramer JD, Givens DJ, Chang CW, et al. The management of salivary fistulas. JAMA Otolaryngol Head Neck Surg. 2023;149 (3):270–7.
32	Original article	Nourissat G, Biau J, Laqueille B, et al. Nutritional therapy during the treatment of head and neck cancer. Oncol Clin Pract. 2018;14 (2):76–84.
33	Systematic review	Huang X, et al. Early oral feeding and its impact on postoperative outcomes in upper gastrointestinal surgery: a systematic review and meta-analysis. Front Surg. 2024;11:123456.
34	Systematic review	Belcastro A, Reed W, Puscas L. The management of salivary fistulas. Semin Plast Surg. 2023;37 (1):4–8. doi:10.1055/s-0042-1759561. PMID: 36776805; PMCID: PMC9911217.

Although these measures are biologically plausible and align with conservative management of established salivary fistulas, formal trials that test dietary restriction as a primary preventive intervention after parotidectomy are lacking. The panel therefore treated available recommendations as expert-opinion-based background information and used them, together with ERAS and salivary fistula literature, as a scaffold for constructing pragmatic, consensus-driven statements.

### Stratification by surgical extent and time

To translate these general principles into a practical tool, the panel formulated a surgery-stratified, time-based framework for diet advancement after parotidectomy (Table 2). Resections were categorised using the ESGS/EMSGS classification, distinguishing limited superficial resections (typically involving lateral levels), isolated resection of the accessory lobe, deep-lobe procedures, and total/subtotal parotidectomy. For each category, typical meal patterns were outlined for the first four postoperative weeks, together with explicit cautions regarding sialagogues and mechanical load (Table 3).

**Table 2:** Key variables for postoperative dietary management after salivary gland surgery.

Variable category	Variable name	Definition/examples	Notes/rationale	Expert priority (1–5)
Patient characteristics	General condition of patient	Overall clinical status, major comorbidities	Determines global risk and overall restrictiveness of diet	1–5
	Age	Chronological age (years)	Frailty and advanced age may require slower progression	1–5
	Nutritional status	BMI, weight loss, sarcopenia, cachexia	Malnutrition indicates need for dietitian input and support	1–5
	Metabolic disorders	Diabetes, endocrine and other metabolic diseases	Poor control can impair wound healing and increase risk	1–5
Tumour/procedure	Tumour size	Maximal diameter (cm)	Larger lesions often require more extensive surgery	1–5
	Tumour location	Gland and anatomical site (e.g., deep vs. lateral, anterior)	Deep or unfavourable sites increase risk of salivary leakage	1–5
	Type/extent of surgery	Procedure name; EMSGS levels resected	Primary driver of duration and intensity of dietary restriction	1–5
Dietary management	Diet type	Liquid, semi-solid, soft, soft-regular, regular	Defines the stepwise advancement pathway	1–5
	Duration of dietary management	Planned days/weeks of modified diet post-operatively	Longer in deep and total resections	1–5
	Number of meals per day	Meal frequency (e.g., 5–6 small meals)	Smaller, frequent portions reduce salivary pressure and discomfort	1–5
	Food consistency	Texture description (pureed, fork-mashable, chewable)	Must match chewing ability and wound stability	1–5
	Recommended foods	Example suitable items at each stage	Focus on soft, non-irritating, protein-rich, energy-dense foods	1–5
	Foods to be avoided	Hard, crunchy, mixed-texture, strongly sialagogue items	Key for preventing sialocele and salivary fistula	1–5
	Additional dietary measures	Oral supplements, protein enrichment, hydration targets	Particularly important in elderly and malnourished patients	1–5
Perioperative management	Compression dressings	Use and duration of pressure dressings at surgical site	Influence tissue support and tolerance of early oral intake	1–5
	Chewing gum	Permitted or not; timing relative to wound stability	Acts as a sialagogue and mechanical stimulus	1–5
	Oral hygiene/dental care	Brushing, mouthwash, dental review	Reduces infection risk and improves comfort	1–5
	Facial rehabilitation	Massage, physiotherapy, cosmetic/functional interventions	Should be coordinated with diet advancement and wound status	1–5
	Pharmacological management	Analgesia, anti-sialagogues, other relevant drugs	Can modulate salivary flow, pain and appetite	1–5
	Antibiotic therapy	Prophylactic or therapeutic regimen	Infection control supports optimal wound healing	1–5

**Table 3:** Postoperative diet by week and parotidectomy extent according to EMSGS.

**Week**	**Total/Subtotal/Deep (I–IV, I-III, III–IV)**	**Limited/superficial (II, V, I–II)**
1st	Pureed/soft bland; 5–6 small meals/day	Strict pureed/very soft bland; avoidance of any sialagogues^*^
2nd	Soft diet; well-cooked, easily chewable foods	Strict soft-bland diet; no hard, crunchy or seedy foods; no sialagogues^*^
3rd	Soft-regular diet (if no swelling or pain)	Soft-regular diet; gradual expansion of food texture; still avoid sialagogues^*^
4th	Regular diet	Regular diet; avoid sialagogues^*^ if possible (especially after prior sialocele-/salivary fistula)

* Sialagogues: sour fruit/juices (citrus), spicy/sour food/beverages, alcohol, coffee.

In total/subtotal or deep procedures, most patients can transition from pureed or very soft bland foods in week 1 to a soft diet with carefully selected soft breads, well-cooked pasta or rice, finely minced meats in sauce, and well-cooked vegetables in week 2. Provided there is no swelling or pain suggestive of salivary complications, a near-normal diet is usually achievable by weeks 3–4, with gradual reintroduction of moderately spicy foods and small quantities of citrus under supervision.

In limited/superficial resections, the protocol is intentionally more conservative. During the first week, patients are advised to consume predominantly pureed and very soft moist foods, with strict avoidance of sour fruit and juices (especially citrus), carbonated drinks, spicy/sour food, and alcohol. A soft-bland diet is maintained into week 2, with only selective addition of soft-regular items and continued prohibition of hard, crunchy, or seedy foods. Transition to a broader soft-regular and then regular diet is generally planned over weeks 3–4, but the timing is adjusted according to clinical stability: persistent swelling, pain, or any evidence of sialocele prompt delay or regression of diet advancement and renewed surgical assessment. In patients with prior sialocele or fistula, strongly sialagogue stimuli (such as citrus, chilli, and strong alcohol) may remain restricted beyond the four-week mark.

**Figure 1:**
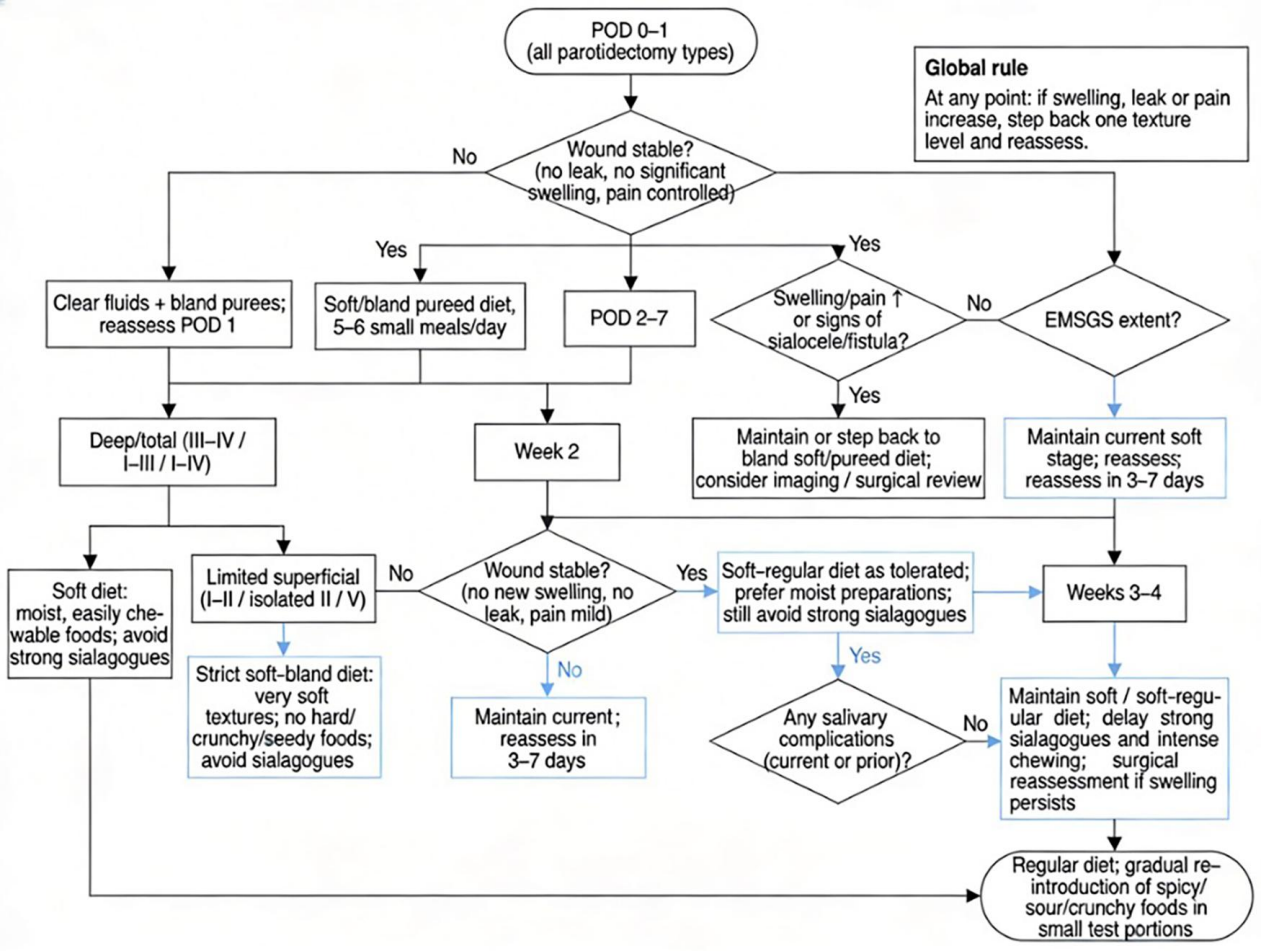
Flowchart of key postoperative decision points and main clinical nodes guiding progression of diet textures after salivary gland surgery.

Across all surgical categories, the guideline specifies decision nodes at postoperative days 0–1, 2–7, week 2, and weeks 3–4. At each time point, diet advancement depends on a combination of factors: wound appearance, presence or absence of fluctuance or swelling, patient-reported pain and chewing difficulty, and any signs of leakage or infection (Table 4 and Figure 1).

**Table 4:** Timeline for diet advancement and oral stimulation after parotidectomy.

**Postoperative period**	**Diet texture focus**	**Oral stimulation strategy**	**Key cautions**
Days 0–1	Clear fluids and bland soft food (purees, cream soups, yogurt); small, frequent portions	Minimal oral stimulation; focus on safe swallowing; without chewing on the operated side	Monitor for new swelling, drain output changes; avoid citrus, carbonated drinks, spicy/sour foods/beverages, alcohol to limit salivary pressure
Days 2–7	Full soft diet (moist, easily chewable foods); maintain high hydration	Gradual reintroduction of chewing, starting on the non-operated side; no deliberate sialagogues	Avoid hard, crunchy textures and strong seasonings; monitor closely for new swelling or fluctuance suggesting sialocele
Week 2	Soft-to-regular diet as tolerated, with preference for moist preparations	Introduction of mild flavour complexity and limited chewing on the operated side if pain and swelling are minimal, small portions	Strongly sialagogue food and beverages remain restricted, particularly after deep or total parotidectomy
Weeks 3–4	Transition to regular diet, guided by EMSGS extent (typically faster for I–IV/I-III, III-IV, slower for II, V, I-II) and healing	Stepwise introduction of oral stimulation (sour, spicy, crunchy) in small test portions	Persistent or increasing swelling warrants regression to a bland soft diet and surgical reassessment; aggressive sialagogues and alcohol may need further delay

Where uncertainty exists, the recommendation is to maintain the current diet level or temporarily revert to a previously tolerated texture rather than to push progression. This conservative, feedback-driven strategy reflects the absence of high-level evidence and the priority placed on preventing salivary complications.

### Patient adherence and education

The expert panel stressed that even the best dietary protocol is ineffective without patient understanding and adherence. Evidence from ERAS-oriented head and neck programmes links compliance with structured nutritional pathways to fewer complications, shorter hospital stays, and better overall recovery. Accordingly, the guideline calls for clear, concise education delivered verbally and in writing, ideally with simple visual or tabular lists of allowed and prohibited foods at each stage. Patients should be informed not only what to eat and avoid, but also why—for example, how certain foods increase salivary pressure or mechanically stress the operative field. In complex cases or in those with poor baseline nutritional status, early involvement of dietitians experienced in head and neck surgery is recommended.

### Key consensus statements (summary)

A set of core statements that underpin the proposed guideline has been proposed: (1) postoperative dietary planning should start with stratification by EMSGS-defined surgical extent, distinguishing limited superficial resections (I–II/isolated II/V) from deep and total/subtotal procedures (III–IV/I–IV/I–III), as limited resections require longer soft/bland regimens and stricter avoidance of sialagogues; (2) across all strata, a soft, non-irritating, low-sialagogue diet should be the default initial pattern, with additional restrictions, such as very low chewing load or avoidance of mixed textures, reserved for extensive surgery and high-risk patients; (3) advancement from soft to more solid textures during days 2–7 should depend on wound stability, absence of increasing pain or swelling, and lack of clinical evidence of sialocele or fistula, with regression to a previously tolerated level advised if any warning signs appear; (4) active oral stimulation through chewing exercises and stepwise re-exposure to sialagogues should be decoupled from basic caloric and fluid intake and considered from weeks 3–4 onwards, provided that local swelling has stabilized and no salivary complications have occurred; and (5) at each decision point (postoperative days 0–1, 2–7, week 2, weeks 3–4), patient-reported tolerance and surgeon assessment of the operative field should jointly determine whether diet texture and stimulation are advanced, maintained, or temporarily regressed.

On this basis, postoperative dietary practices after parotidectomy were formalised into structured, surgery-stratified regimens and a stepwise advancement protocol with predefined assessment time points at days 2, 7, 14, and 28. While inherently provisional, these recommendations provide a clear, implementable framework that can be adopted, audited, and refined in different settings. They also identify specific areas where prospective studies, including pragmatic trials and implementation research, are urgently needed to convert expert-driven practice into truly evidence-based dietary care after parotidectomy.

## Discussion

This consensus statement addresses a long-standing gap in postoperative care, namely the lack of evidence based dietary guidance after parotid gland surgery despite the substantial incidence of sialocele and salivary fistula reported in contemporary series and systematic reviews. Available data consistently show that more conservative resections are associated with higher rates of salivary leakage, secondary infection and wound related morbidity, supporting the view that surgical extent and technical complexity should be primary determinants of postoperative dietary intensity and timing. In this context, the proposed recommendations formalise current best practice by linking softer, low sialagogue diets and slower texture progression to higher risk procedures, while allowing earlier advancement after limited superficial resections when the wound is clinically stable. In keeping with these constraints, the present guideline deliberately integrates peer reviewed evidence with practice derived materials and structured expert opinion rather than claiming to offer a purely evidence based protocol in the strict sense; to enhance transparency, the revised manuscript explicitly distinguishes, where possible, between recommendations primarily supported by peer reviewed studies and those grounded mainly in institutional leaflets, patient information sheets and expert synthesis, and Supplementary Table S1 provides a structured overview of the dominant source type for each key recommendation together with a qualitative assessment of how the proposals would change if only peer reviewed data were considered (4, 10, 11, 17, 23, 24).

This stratified approach is congruent with broader perioperative nutrition and ERAS-oriented literature in head and neck surgery, which emphasises tailoring route, texture, and energy density of nutrition to the degree of anatomical disruption and fistula risk rather than applying uniform protocols. It also takes into account intraoperative manoeuvres that modify ductal outflow or create multiple planes of parenchymal disruption: techniques such as multiple parenchymal ligations or main duct ligation appear to increase vulnerability to intraglandular saliva extravasation, justifying stricter short-term restriction of sialagogues and a more conservative, stepwise pattern of diet advancement until wound stability is assured (10, 15, 16, 20).

From a mechanistic standpoint, these recommendations are consistent with experimental data showing that both mastication and gustatory stimulation substantially increase parotid salivary flow, particularly when chewing natural foods and sour, acid-containing items. Chewing- and taste-induced surges in secretion are likely to transiently raise intraductal and intraparenchymal pressure within the recently operated gland, especially when multiple planes of dissection or ductal manipulations are present. In this context, temporarily limiting intense chewing and strongly sialagogue stimuli provide a biologically plausible means of reducing saliva extravasation into dissected tissues and thereby lowering the risk of sialocele and salivary fistula (10, 29–32).

At first sight, the stricter short term dietary regimen proposed for limited/superficial resections may appear counterintuitive, as these procedures involve less extensive anatomical disruption than deep lobe or total parotidectomy. However, contemporary series and collective experience from high volume centres suggest that conservative superficial resections—especially those involving mid gland and anterior tumours—can exhibit relatively high rates of sialocele and minor salivary leakage, likely because a large volume of functioning parotid tissue and intact ductal arborisation is left in continuity with shallow dissected planes. From a pathophysiological perspective, this configuration provides fertile ground for saliva extravasation when chewing forces and strong sialagogue stimuli generate transient peaks in intraglandular pressure, which is why the panel favoured a more conservative regimen in the early weeks after limited/superficial surgery. Supplementary Table S2 summarises this clinical and mechanistic rationale in a structured manner and relates it directly to the week-by-week advancement schedule, while explicitly acknowledging that, in the absence of prospective dietary trials, this pattern is primarily grounded in biological plausibility and observed complication patterns rather than controlled comparisons (15, 19, 24).

A further key element is the recognition that patient adherence is critical for translating these principles into improved outcomes. Experience from ERAS-based programmes indicates that adherence to structured nutritional pathways, including early planned intake and continuation of supplements, correlates with fewer complications and shorter hospital stays, whereas non-compliance—driven by inadequate counselling, low health literacy, or psychosocial barriers—may compromise wound healing even in the absence of parotid-specific trials. For this reason, the consensus underscores the need for clear, comprehensible education, supported by written and visual materials, and early involvement of dietitians in patients with poor baseline nutritional status or complex needs (27).

Finally, the guideline explicitly frames proposed postoperative days for diet advancement as flexible decision points rather than rigid rules. At each node (days 0–1, 2–7, week 2, weeks 3–4), the choice to maintain, advance or regress diet texture is contingent on wound appearance, pain, swelling and any clinical signs of sialocele or fistula, thereby transforming fixed timetables derived from experiential practice into stratified, surgery dependent pathways. In the absence of high level comparative studies, this combination of pathophysiological rationale, structured experience from reference centres and transparent, surgery stratified algorithms offer a pragmatic basis for standardising postoperative diet after parotidectomy and clearly delineates priorities for future prospective and interventional research. Importantly, when the recommendations are viewed through the lens of peer reviewed evidence alone, the overarching principles remain unchanged—a cautious, soft, low sialagogue diet in the early postoperative phase, stratified by surgical extent and tailored to wound stability, is consistent with both the salivary fistula literature and broader perioperative nutrition data—whereas what is lost, as detailed in Supplementary Table S1, is mainly the fine granularity of the week by week timetable and the concrete lists of example foods, which necessarily derive from grey literature and expert practice rather than from comparative dietary trials.

This consensus statement has several important limitations that should be acknowledged. First, there are no prospective, adequately powered clinical trials specifically evaluating postoperative dietary regimens after parotidectomy, and from a practical and ethical standpoint it is difficult to envisage randomising patients to a truly unrestricted diet arm in the early postoperative period, so the proposed recommendations cannot be regarded as evidence based in the strictest sense and should be viewed as hypothesis generating. Second, although the guideline is evidence informed, many statements still rely on structured expert opinion, which is inherently vulnerable to selection bias and the prevailing beliefs of surgeons from high volume reference centres rather than reflecting broader international practice. Third, the underlying literature is highly heterogeneous, with substantial variation in surgical techniques, definitions and reporting of salivary complications, local postoperative routines and even customary dietary patterns between different regions and countries, which limits the generalisability of any unified protocol. Fourth, the targeted review necessarily incorporated patient information leaflets, institutional postoperative instructions and curated internet based resources alongside peer reviewed studies; while these materials offer valuable insight into real world practice, they are not formally appraised or standardised and may perpetuate untested assumptions, and their relative contribution to individual recommendations is therefore made explicit in Supplementary Table S1. Finally, the recommendations have not yet been prospectively implemented and audited in diverse settings, so their feasibility, acceptability and impact on clinical outcomes remain uncertain and should be formally evaluated in future multicentre prospective studies. Postoperative dietary management after parotidectomy remains guided by low-level evidence and heterogeneous local practice rather than robust clinical trials. Our expert-panel recommendations offer a pragmatic, surgery-stratified framework that links diet texture, timing, and sialagogue restriction to salivary fistula risk and wound stability. These consensus-based protocols should be implemented cautiously, audited systematically, and refined through future prospective and implementation studies to establish truly evidence-based postoperative nutrition pathways in salivary gland surgery.

In summary, in the absence of high-level comparative data, this consensus provides a transparent, surgery stratified framework for postoperative diet after parotidectomy that couples soft, low sialagogue regimens with clearly defined decision points linked to wound stability. It is intended as a pragmatic, auditable starting point for standardizing care and for designing future prospective and implementation studies that will ultimately transform expert driven practice into truly evidence based postoperative nutrition pathways.

## References

[1] WHO Global Cancer Observatory fact - sheet. Available online at: https://gco.iarc.who.int/media/globocan/factsheets/cancers/2-salivary-glands-fact-sheet.pdf

[2] Talati V Brown HJ Losenegger T Revenaugh P Al-Khudari S. Patient safety and quality improvements in parotid surgery. World J Otorhinolaryngol–Head and Neck Surg. (2022) 8:133–38. 10.1002/wjo2.50 35782399 PMC9242422

[3] Piwowarczyk K Bartkowiak E Klimza H Greczka G Wierzbicka M. Review and characteristics of 585 salivary gland neoplasms from a tertiary hospital registered in the Polish national Major salivary gland benign tumors registry over a period of 5 years: a prospective study. Otolaryngol Pol. (2020) 74:1–6. 10.5604/01.3001.0014.1261 34550095

[4] Stathopoulos P Igoumenakis D Smith WP. Partial superficial, superficial, and total parotidectomy in the management of benign parotid gland tumors: a 10-year prospective study of 205 patients. J Oral Maxillofac Surg. (2018) 76:455–59. 10.1016/j.joms.2017.06.018 28704636

[5] Belcastro A Reed W Puscas L. The management of salivary fistulas. Semin Plast Surg. (2023) 37:4–8. 10.1055/s-0042-1759561 36776805 PMC9911217

[6] Lhotský R. Diet after reduction of the saliva gland (2024) Available at: Available online at: https://www.radeklhotsky.cz/en/diet-after-reduction-of-the-submandibular-gland/

[7] Tibrewal S. Diet plan after salivary gland surgery: what foods to eat and avoid (2023). Available online at: https://www.drsuniltibrewal.com/diet-plan-foods-to-eat-avoid-after-salivary-gland-surgery/

[8] Witt RL. The incidence and management of siaolocele after parotidectomy. Otolaryngol Head Neck Surg. (2009) 140:871–4. 10.1016/j.otohns.2009.01.021 19467406

[9] Gahir D Clifford N Yousefpour A Avery C. A novel method of managing persistent parotid sialocele. Br J Oral Maxillofac Surg. (2011) 49:491–2. 10.1016/j.bjoms.2010.07.019 21177006

[10] Parekh D Glezerson G Stewart M Esser J Lawson HH. Post-traumatic parotid fistulae and sialoceles. A prospective study of conservative management in 51 cases. Ann Surg. (1989) 209:105–11. 10.1097/00000658-198901000-00015 2910210 PMC1493871

[11] Britt CJ Stein AP Gessert T Pflum Z Saha S Hartig GK. Factors influencing sialocele or salivary fistula formation postparotidectomy. Head Neck. (2017) 39:387–91. 10.1002/hed.24564 27550745

[12] Marchese-Ragona R De Filippis C Staffieri A Restivo DA Restino DA. Parotid gland fistula: treatment with botulinum toxin. Plast Reconstr Surg. (2001) 107:886–7. 10.1097/00006534-200103000-00048 11314663

[13] Maharaj S Mungul S Laher A. Botulinum toxin A is an effective therapeutic tool for the management of parotid sialocele and fistula: a systematic review. Laryngoscope Investig Otolaryngol. (2020) 5:37–45. 10.1002/lio2.350 32128429 PMC7042652

[14] Larian B Azizzadeh B. Parotidectomy Recovery Guidelines.. Beverly Hills, CA: Center for Advanced Parotid & Facial Nerve Surgery (2025). Available online at: https://www.parotidsurgerymd.com/micro-parotidectomy/parotidectomy-recovery-guidelines/

[15] Liu Y Yuan W Sun H Su M Kong X Huang X. Predictors of sialocele or salivary Fistula after partial superficial parotidectomy for benign parotid tumor: a retrospective study. J Oral Maxillofac Surg. (2022) 80:327–32. 10.1016/j.joms.2021.09.013 34662554

[16] Yang X Ge S Tao Y Li J Shang W Song K. Assessment of the observation management of sialocele after partial superficial parotidectomy. Oral Dis. (2023) 29:996–1004. 10.1111/odi.14079 34773330

[17] Zeng Q Sheng M Ouyang S Liu B. Influence of inflammatory and nutritional indices on post-parotidectomy salivary fistula or sialocele in benign tumors. J Oral Maxillofac Surg Med Pathol. (2026) 38:36–42. 10.1016/j.ajoms.2025.06.015

[18] Quer M Guntinas-Lichius O Marchal F Vander Poorten V Chevalier D León X. Classification of parotidectomies: a proposal of the European salivary gland society. Eur Arch Otorhinolaryngol. (2016) 273:3307–12. 10.1007/s00405-016-3916-6 26861548

[19] Lambiel S Dulguerov N Courvoisier DS Dulguerov P. Minor parotidectomy complications: a systematic review. Laryngoscope. (2021) 131:571–9. 10.1002/lary.28912 32678921

[20] Sadok N Bastian T Voss N Stähr K Arweiler-Harbeck D Lang S. Comparative analysis of Fistula development after parotid gland surgery: lateral parotidectomy versus extracapsular dissection technique. Clin Otolaryngol. (2024) 49:793–800. 10.1111/coa.14213 39145398

[21] Reerds STH Hey SY van den Hoogen FJA Takes RP Ganesh V Marres HAM. Outpatient parotidectomy with or without the use of a post-operative drain: a retrospective bi-institutional study. Clin Otolaryngol. (2023) 48:430–5. 10.1111/coa.14028 36585381

[22] Nielsen CF Riis CBS Christensen ALB Mirz F Reinholdt KB Ovesen T. Superficial parotidectomy: impact of postoperative drainage. Ear Nose Throat J. (2022) 101:105–9. 10.1177/0145561320942380 32744902

[23] Galli A Tulli M Vella A Familiari M Giordano L Bondi S. The importance of the patient’s perspective in function-sparing parotid surgery for benign neoplasms: clinical reappraisal. Acta Otorhinolaryngol Ital. (2021) 41:410–18. 10.14639/0392-100X-N1463 34734576 PMC8569663

[24] Alabdulqader A Alessa M Aldhahri S Alqahtani K. Factors associated with postoperative complications in patients who underwent parotidectomy: a retrospective study. Ear Nose Throat J. (2024) 1455613241244656. 10.1177/01455613241244656 38721821

[25] Bluman LG Mosca L Newman N Simon DG. Preoperative smoking habits and postoperative pulmonary complications. Chest. (1998) 113:883–89. 10.1378/chest.113.4.883 9554620

[26] Kaki PC Patel AM Brant JA Cannady SB Rajasekaran K Brody RM. Hypoalbuminemia and postoperative outcomes following major salivary gland resection. Laryngoscope Investig Otolaryngol. (2025) 10:e70107. 10.1002/lio2.70107 40012621 PMC11863205

[27] Bertazzoni G Testa G Tomasoni M Mattavelli D Del Bon F Montalto N. The enhanced recovery after surgery (ERAS) protocol in head and neck cancer: a matched-pair analysis. Acta Otorhinolaryngol Ital. (2022) 42:325–33. 10.14639/0392-100X-N2072 36254650 PMC9577693

[28] Palacios V Holley A Park A. Management of a salivary fistula following removal of mandibular distractors in a neonate. Am J Otolaryngol. (2023) 44:103720. doi: 10.1016/j.amjoto.2022.103720 36493470

[29] Lazaridou M Iliopoulos C Antoniades K Tilaveridis I Dimitrakopoulos I Lazaridis N. Salivary gland trauma: a review of diagnosis and treatment. Craniomaxillofac Trauma Reconstr. (2012) 5:189–96. 10.1055/s-0032-1313356 24294401 PMC3577598

[30] Mackie DA Pangborn RM. Mastication and its influence on human salivary flow and alpha-amylase secretion. Physiol Behav. (1990) 47:593–5. 10.1016/0031-9384(90)90131-m 2359773

[31] Watanabe S Dawes C. A comparison of the effects of tasting and chewing foods on the flow rate of whole saliva in man. Arch Oral Biol. (1988) 33:761–4. 10.1016/0003-9969(88)90010-6 3252776

[32] Feller RP Sharon IM Chauncey HH Shannon IL. Gustatory perception of sour, sweet, and salt mixtures using parotid gland flow rate. J Appl Physiol. (1965) 20:1341–44. 10.1152/jappl.1965.20.6.1341

