# Supplementary material for: Postoperative dietary management after parotid gland surgery in the absence of high-level evidence: consensus-based guidelines and a stepwise protocol for diet advancement
Source: Front Surg. 2026 Jun 26;13:1849468. doi: 10.3389/fsurg.2026.1849468 (PMC13350029; doi:10.3389/fsurg.2026.1849468)
Supplement: Supplementary file 1 [file Table1.docx]

## Supplementary table 1. Source type and impact of restricting recommendations to peer‑reviewed evidence.

| **Domain / item** | **Dominant source type (peer‑reviewed vs grey)** | **Examples of key sources (illustrative)** | **Role in current recommendations** | **Effect if restricted to peer‑reviewed evidence only** |
| --- | --- | --- | --- | --- |
| **Existence of an evidence gap in postoperative diet after parotidectomy** | Peer‑reviewed | Review and original articles on salivary fistula/sialocele and perioperative nutrition | Establishes that no prospective or randomized trials specifically test dietary regimens after parotid surgery, justifying an expert‑informed approach | Unchanged: the recognition of a major evidence gap and need for pragmatic guidance remains fully supported |
| **Association between salivary leakage and wound‑related morbidity** | Peer‑reviewed | Original studies on sialocele/fistula rates and outcomes | Underpins the need to consider diet as a potential modifiable factor in postoperative care | Unchanged: the importance of preventing salivary complications remains strongly evidence‑based |
| **General principles of soft, bland, low‑sialagogue diet in early postoperative phase** | Combined | Belcastro 2023; Cramer 2023; head and neck nutrition literature; institutional leaflets | Supports cautious diet textures and restriction of strong sialagogues in the early postoperative period | Core principle preserved, but examples and timing become less granular |
| **Detailed lists of recommended and contraindicated foods** | Grey | Institutional postoperative leaflets; patient diet booklets; parotid surgery websites | Provide practical, patient‑friendly examples (textures, specific foods, beverages) at each protocol step | Markedly weakened: only generic “soft / low‑irritant diet” statements would remain, with few concrete examples |
| **Week‑by‑week timetable for diet advancement by EMSGS extent** | Grey (expert synthesis) | Curated postoperative instructions from multiple high‑volume centres; expert consensus | Produces the structured, surgery‑stratified advancement schedule in Tables 3–4 | Substantially reduced detail: surgery‑stratified “early / intermediate / late” phases could be outlined, but without precise timing |
| **Emphasis on hydration and energy‑dense intake** | Peer‑reviewed | Head and neck ERAS and nutritional therapy articles | Justifies prioritising adequate caloric and fluid intake despite texture restrictions | Unchanged: the need for sufficient energy and hydration remains strongly evidence‑based |
| **Caution with citrus, carbonated drinks, spicy foods and alcohol** | Combined | Fistula management literature; multiple institutional protocols | Provides the basis for recommending temporary avoidance of high‑sialagogue stimuli | Principle preserved, but the duration and exact spectrum of restricted items would be less precisely defined |
| **Use of dietitian support in high‑risk or malnourished patients** | Peer‑reviewed | Nutritional therapy guidelines in head and neck oncology | Supports involving dietitians in complex or nutritionally vulnerable patients | Unchanged: remains fully supported |
| **Surgery‑stratified logic (limited/superficial vs deep/total resections)** | Combined | Observational series on complication patterns; expert experience | Justifies tailoring the stringency and duration of diet restrictions to EMSGS‑defined extent | Qualitative stratification preserved; the exact week‑by‑week schedules become less firmly anchored |
| **Overall structure of the stepwise, surgery‑stratified protocol** | Combined | All the above, integrated | Produces a coherent, implementable pathway for clinical use | Framework remains, but with lower granularity and fewer concrete examples of food items and time points |

This table distinguishes, for each major component of the proposed guideline, whether it is primarily supported by peer‑reviewed evidence, by grey literature, or by a combination of both, and qualitatively describes how the recommendations would change if restricted to peer‑reviewed sources alone.
