# Supplementary material for: Postoperative dietary management after parotid gland surgery in the absence of high-level evidence: consensus-based guidelines and a stepwise protocol for diet advancement
Source: Front Surg. 2026 Jun 26;13:1849468. doi: 10.3389/fsurg.2026.1849468 (PMC13350029; doi:10.3389/fsurg.2026.1849468)
Supplement: Supplementary file 2 [file Table2.docx]

## Supplementary table 2. Rationale for a stricter short‑term dietary regimen after limited/superficial resections

| **Aspect / question** | **Limited/superficial resections  (e.g. EMSGS levels I–II, V)** | **Deep or total/subtotal resections (e.g. levels III–IV, I–IV, I–III)** | **Implication for dietary stringency** |
| --- | --- | --- | --- |
| **Residual gland volume and secretory capacity** | Large volume of functioning parotid parenchyma preserved; ductal arborisation largely intact | Substantial parenchymal reduction; ducts may be ligated, divided or re‑routed | Higher baseline and stimulated saliva production in close proximity to fresh dissected planes favours stricter early diet in limited cases |
| **Relationship between ducts and dissected tissue planes** | Multiple small ducts and ductules remain in continuity with shallow dissection planes and skin–SMAS flap | Deeper dissection planes may be better isolated or obliterated by reconstruction and fibrosis | Greater opportunity for saliva to extravasate into superficial tissues after limited resections |
| **Mechanical load on the operative field** | Thinner, soft‑tissue coverage; less intrinsic support; chewing forces more directly transmitted to wound | Deeper bed, often with more robust multilayer closure and regional soft‑tissue bulk | Early hard or mixed‑texture foods may more easily destabilise closure in superficially dissected fields |
| **Effect of mastication and gustatory stimulation** | Intact secretory apparatus responds vigorously to chewing and sialagogues, increasing intraglandular pressure | Secretory response may be attenuated by parenchymal reduction and ductal interruption in some cases | Transient pressure peaks are more likely to cause micro‑leakage into dissected planes after limited/superficial resections |
| **Observed pattern of salivary complications (clinical experience and series)** | Paradoxically higher rates of sialocele and minor salivary leakage reported in some series of “conservative” resections | Salivary complications certainly present, but not consistently higher than in limited resections | Favouring more conservative early diet in limited/superficial surgery despite apparently smaller anatomical disruption |
| **Short‑term dietary recommendations (Weeks 1–2)** | Week 1: strict puréed/very soft bland diet; avoidance of hard, crunchy and seedy foods; absolute restriction of strong sialagogues (citrus, spicy foods, carbonation, alcohol). Week 2: continuation of a soft‑bland diet with only cautious introduction of soft‑regular items if the wound is completely stable. | Week 1: pureed/soft bland diet; introduction of soft foods as tolerated. Week 2: progression to a broader soft diet with earlier introduction of soft‑regular textures in the absence of swelling or pain. | Short‑term regimen is intentionally stricter and longer in limited/superficial resections to minimise salivary surges and mechanical stress on vulnerable superficial planes |
| **Medium‑term dietary recommendations (Weeks 3–4)** | Stepwise expansion of textures and flavours, contingent on complete absence of swelling, fluctuance or pain; strong sialagogues may remain restricted beyond week 4 in patients with prior sialocele/fistula. | Transition to regular diet by weeks 3–4 in most patients, with gradual re‑introduction of spicy foods and small amounts of citrus under supervision. | The stricter early regimen is gradually relaxed once wound stability is assured, maintaining a more cautious stance only in patients with complications |
| **Pathophysiological rationale in one sentence** | High residual secretory capacity, preserved ductal continuity and shallow dissected planes create a permissive environment for saliva extravasation under conditions of intense mastication and sialagogue exposure. | Parenchymal reduction, duct manipulation and deeper dissection may, in selected cases, partially limit salivary pressure at the wound interface. | Justifies the apparently counterintuitive choice of a stricter short‑term dietary regimen in limited/superficial resections |

This table summarises the clinical and pathophysiological rationale for applying a comparatively stricter short‑term dietary regimen after limited/superficial resections than after some deep or total parotid procedures, highlighting how residual gland function, ductal continuity and superficial dissection planes interact with mastication and sialagogue exposure to influence sialocele and salivary fistula risk.
